# Interaction Effects of AFB1 and MC-LR Co-exposure with Polymorphism of Metabolic Genes on Liver Damage: focusing on SLCO1B1 and GSTP1

**DOI:** 10.1038/s41598-017-16432-z

**Published:** 2017-11-23

**Authors:** Xiaohong Yang, Wenyi Liu, Hui Lin, Hui Zeng, Renping Zhang, Chaowen Pu, Lingqiao Wang, Chuanfen Zheng, Yao Tan, Yang Luo, Xiaobin Feng, Yingqiao Tian, Guosheng Xiao, Jia Wang, Yujing Huang, Jiaohua Luo, Zhiqun Qiu, Ji-an Chen, Liping Wu, Lixiong He, Weiqun Shu

**Affiliations:** 1 Department of Environmental Hygiene, College of Preventive Medicine, Third Military Medical University, Chongqing, 400038 China; 2 Department of Tropical Epidemiology, College of Preventive Medicine, Third Military Medical University, Chongqing, 400038 China; 3 The Center for Disease Control and Prevention in Fuling District, Chongqing, 408000 China; 4 Center for Nanomedicine, Southwest Hospital, Third Military Medical University, Chongqing, 400038 China; 5 Institute of Hepatobiliary Surgery, Southwest Hospital, Third Military Medical University, Chongqing, 400038 China; 6 College of Life Science and Engineering, Chongqing Three Gorges University, Wanzhou, Chongqing, 404100 China; 7 Department of Health Education, College of Preventive Medicine, Third Military Medical University, Chongqing, 400038 China

## Abstract

AFB1 and MC-LR are two major environmental risk factors for liver damage worldwide, especially in warm and humid areas, but there are individual differences in health response of the toxin-exposed populations. Therefore, we intended to identify the susceptible genes in transport and metabolic process of AFB1 and MC-LR and find their effects on liver damage. We selected eight related SNPs that may affect liver damage outcomes in AFB1 and MC-LR exposed persons, and enrolled 475 cases with liver damage and 475 controls of healthy people in rural areas of China. The eight SNPs were genotyped by PCR and restriction fragment length polymorphism. We found that SLCO1B1 (T521C) is a risk factor for liver damage among people exposed to high AFB1 levels alone or combined with MC-LR, and that GSTP1 (A1578G) could indicate the risk of liver damage among those exposed to high MC-LR levels alone or combined with high AFB1 levels. However, GSTP1 (A1578G) could reduce the risk of liver damage in populations exposed to low MC-LR levels alone or combined with high AFB1 levels. In conclusion, SLCO1B1 (T521C) and GSTP1 (A1578G) are susceptible genes for liver damage in humans exposed to AFB1 and/or MC-LR in rural areas of China.

## Introduction

Aflatoxins, HBV and microcystins are three risk factors for liver cancer in the world. Aflatoxins and microcystins are environmental hazards in our daily life, among which aflatoxins are food contaminants and microcystins water pollutants, and can cause liver damage at a low level with long-term accumulation.

Aflatoxins are secondary metabolites produced by *Aspergillus flavus* and *Aspergillus parasiticu*s naturally occurring under warm and moist environmental conditions, and are common contaminants of a number of staple foods including maize, groundnuts, rice and sorghum during growth, harvest and storage. The toxins are widely distributed and can pose serious public health hazards to humans due to their toxic, teratogenic, mutagenic, and highly carcinogenic properties^1^. Early research has showed that dietary aflatoxin exposure could increase the morbidity and mortality of HCC^2^. There are four major toxins, namely AFB1, AFB2, AFG1 and AFG2. Among them, AFB1 is most often found in contaminated food, and has the highest hepatocarcinogenic risk. The metabolism of aflatoxin is mainly through liver^3,4^, and the major human cytochrome p450 enzymes involved in its metabolism are CYP3A4, 3A5, 3A7 and 1A2^4^. AFB1 is metabolized to a reactive epoxide (aflatoxin-8,9-epoxide, AFBO) catalyzed by CYP1A2 and CYP3A4 in humans^5,6^. GSH and GST participate in the protection of tissue from deleterious effects of AFB1 intoxication including carcinogenetic effects^7–9^. In humans and most animals, the major route of AFB1 detoxification is via conjugation of AFBO to endogenous GSH by the classical detoxification glutathione S-transferases (GSTs)^10^.

The cyanobacteria bloom is a principal water environment problem in warm areas, and it is the same case with the water bodies of the Three Gorges Reservoir Region in Chongqing, China^11^ as well as many freshwater lakes of China, such as Tai Lake, Dianchi Lake and Chao Lake^12–14^. Microcystins (MCs) are a class of biologically active single cyclic heptapeptides mainly produced by the freshwater algae Microcystis aeruginosa^15^, among which, microcystin-LR (MC-LR) is one of the most toxic variants in freshwater environment of China^16^. MC-LR is taken up into the hepatocyte via organic anion transporting polypeptides (OATP/Oatp)^17^, including confirmed liver-specific human OATP1B1 and OATP1B3, and mouse Oatp1b2 (mOatp1b2)^18–22^. The coding genes of OATP1B1 and OATP1B3 are SLCO1B1 and SLCO1B3, respectively. The accepted pathway of MC-LR detoxification in liver and excretion in urine is GSH conjugation through nucleophilic reaction of the thiol to the α, β-unsaturated carbonyl of the Mdha moiety catalyzed by GSTs^23–25^ and transported to the kidneys and intestines for excretion. CYP2E1 might be a potential source responsible for ROS generation by MC-LR^26–28^.

There are many gene polymorphisms of OATP, GSTs and CYP450 affecting the change of protein function or quantity reported by the literatures. We deduced that the gene polymorphism of transport and metabolic pathways of AFB1 and MC-LR could affect the transport and metabolism of the two toxins, leading to differences in the individual sensitivity among AFB1 and MC-LR exposed populations.

Chongqing is located in Southwest China, with Yangtze River crossing from west to east. Climate of Chongqing is mild, and belongs to the humid sub-tropical monsoon climate, with an average temperature of 16~18 °C and an average annual precipitation of 1000~1350 mm in most of the area. The warm and humid environment of Chongqing is a key premise leading to the occurrence of AFB1 in food and MC-LR in water.

There is no study reporting the joint action of gene polymorphisms of transport and metabolic pathways with the two toxins on liver damage. Eight SNPs were selected in transport and metabolic pathways, including SLCO1B1 (T521C, rs4149056), SLCO1B3 (T334G, rs4149117), GSTT1 (−/+, rs4025935), GSTM1 (−/+, rs71748309), GSTA1 (C69T, rs3957357), GSTP1 (A1578G, rs1695), CYP2E1 (C1019T, rs2031920) and CYP3A4 (A13871G, rs55951658). We took Chongqing as the study field to find the susceptible genes in transport and metabolic process of AFB1 and MC-LR to provide a scientific basis for risk management of food and water.

## Results

### Demographic and clinical characteristics of the study population

In the final analysis, we recruited 950 adults (475 cases and 475 controls) from the cross-sectional investigation (Table 1), including 310 males and 165 females both in cases and in controls, and the proportion of males was 65.3%. The average ages of the cases and controls were 60.61 and 61.16 years old, respectively. The local people in our research had long time exposure to AFB1 and MC-LR. The average exposure years of local residence were 51.19 years in cases, and 52.51 years in controls. There were no significant differences between cases and controls in terms of distribution of sex, age, daily water intake, residence, income, alcohol drinking status, smoking, passive smoking and tea drinking habit as a result of individual matching (*P* > 0.05) in groups of adults, while there were significant differences between cases and controls in BMI (*P* < 0.05). The clinical characteristics (ALT, AST) were significantly higher in cases than in controls of the target population. These results suggested that data of cases with liver damage were comparable with the data of controls.

**Table 1:** Demographic and clinical characteristics of the study population.

Characteristics	Cases (475)	Controls (475)	P-value^a^
Sex, n(%) Male	310 (65.3)	310 (65.3)	1.000^b^
Female	165 (34.7)	165 (34.7)	
Age (year, M ± SD)	60.61 ± 11.68	61.16 ± 11.62	0.412^c^
BMI (M ± SD)	24.5 ± 3.60	23.63 ± 3.36	0.001^c^
Daily water intake (L, M ± SD)	1.39 ± 0.891	1.51 ± 2.062	0.252^c^
Local Residence (years, M ± SD)	51.19 ± 19.21	52.51 ± 19.58	0.294^c^
Income (ten thousands RMB, M ± SD)	2.68 ± 5.92	2.64 ± 3.46	0.901^c^
Drinking status, n(%) Yes	55 (13.2)	65 (15.4)	0.461^b^
No	362 (86.8)	356 (84.6)	
Smoking, n(%) Yes	77 (18.4)	78 (18.3)	0.497^b^
No	341 (81.6)	349 (81.7)	
Passive smoking, n(%) Yes	212 (47.7)	8 (47.1)	0.577^b^
No	232 (52.3)	9 (52.9)	
Tea drinking habit, n(%) Yes	55 (12.9)	57 (13.3)	0.700^b^
No	373 (87.1)	370 (86.7)	
ALT (U/L, M ± SD)	33.56 ± 32.97	16.32 ± 7.02	0.000^c^
AST (U/L, M ± SD)	38.16 ± 32.19	20.99 ± 6.83	0.000^c^
Serum Level of MC-LR (μg/L, M ± SD)	0.282 ± 0.231	0.268 ± 0.202	0.718^c^
Serum Level of AFB1 (μg/L, M ± SD)	3.132 ± 2.248	2.935 ± 2.285	0.217^c^

Note: ^a^Comparing Cases with Controls. ^b^From chi-square test. ^c^From t test.

### Effects of AFB1 and MC-LR exposure on liver damage

Information on exposure to AFB1 and MC-LR of the study population is shown in Table 1. We found that the cases of adults (3.123 ng/L) had a higher mean serum level of AFB1 than controls (2.935 ng/L), but no differences (*P* > 0.05) were found. For serum MC-LR in the study, there were no differences between cases and controls (*P* > 0.05).The mean serum levels of MC-LR were 0.282 ng/L and 0.268 ng/L in cases and controls, respectively.

### Effects of SLCO1B1 and GSTP1 polymorphisms on liver damage

The genotype and distribution of control group were consistent with those expected from Hardy-Weinberg’s equilibrium. Among the eight SNPs, only SLCO1B1 (T521C) and GSTP1 (A1578G) were observed to modify liver damage of cases and controls (Table 2). We analyzed the risk of genotypes for gene SLCO1B1 (T521C) in cases and controls, and found that the OR of genotype TC *versus* genotype TT was 0.743 (95%CI: 0.560-0.985), and the *P* value is 0.023. The frequencies of mutation genotypes (TC and CC) and allele (C) were 63.3% and 35.6% in cases, respectively; the corresponding frequencies for SLCO1B1 (T521C) were 69.6% and 38.7% in controls. For GSTP1 between the two groups, there were significant differences between people carrying GG *versus* those carrying AA in case and control groups. The OR was 1.367 (95%CI: 1.017-1.837), and the *P* value was 0.023. Risk value of liver damage for the mutation genotype with GSTP1 (A1578G)-AG/GG was 1.429 (95%CI: 1.029-1.892) *versus* wild genotype GSTP1(A1578G)-AA; whereas risk value was 1.375(95%CI: 1.123-1.685) for GSTP1 (A1578G)-A allele *versus* G allele. For the other six SNPs, SLCO1B3 (T334G), GSTT1 (−/+), GSTM1 (−/+), GSTA1 (C69T), CYP2E1 (C1019T) and CYP3A4 (A13871G), we found no statistical differences in cases and controls of the target population (Supplementary material). These results suggested that the risk of liver damage may be associated with SLCO1B1 (T521C) and GSTP1 (A1578G) among people exposed to AFB1 or/and MC-LR for a long time.

**Table 2:** Genotype and allele frequencies of SLCO1B1 (T521C) and GSTP1 (A1578G) in cases and controls.

Gene	Genotype	Cases n(%)	Controls n(%)	OR (95%CI)	P-value^a,b^
SLCO1B1	TT	164 (36.7)	141 (30.4)	ref	
	TC	248 (55.5)	287 (61.9)	0.743 (0.560–0.985)	0.023
	CC	35 (7.8)	36 (7.8)	0.836 (0.499–1.402)	0.291
	TC/CC	283 (63.3)	323 (69.6)	0.753 (0.572–0.993)	0.410
	T	576 (64.4)	569 (61.3)	ref	
	C	318 (35.6)	359 (38.7)	0.875 (0.723–1.058)	0.092
GSTP1	AA	318 (66.9)	353 (74.3)	ref	
	AG	24 (5.1)	14 (2.9)	1.903 (0.968–3.742)	0.042
	GG	133 (28.0)	108 (22.7)	1.367 (1.017–1.837)	0.023
	AG/GG	157 (33.1)	122 (25.7)	1.429 (1.029–1.892)	0.008
	A	660 (69.5)	720 (75.8)	ref	
	G	290 (30.5)	230 (24.2)	1.375 (1.123–1.685)	0.001

Note: ^a^Comparing Cases with Controls. ^b^From chi-square test.

### Interaction of SLCO1B1 (T521C) with AFB1 or MC-LR on liver damage

Table 3 shows the interaction of SLCO1B1 (T521C) with AFB1 or MC-LR alone on human liver damage. When people were exposed to a low AFB1 level, there was no interaction between SLCO1B1 (T521C) and human liver damage. However when people were exposed to a high AFB1 level, liver damage was easier to occur to people carrying the mutation genotypes (TC and CC), compared to those carrying wild genotype (TT). The OR was 2.578(95%CI: 1.715-3.876, *P* < 0.05), and its frequencies of mutation genotypes were 43.9% and 23.3% in cases and controls, respectively; For allele, risk value of liver damage with SLCO1B1-C was 1.580(95%CI: 1.200-2.080, *P* < 0.05), and the frequencies of mutation allele C were 68.2% and 57.2% in cases and controls, respectively. The above results showed that when exposed to a high AFB1 level (≥2.93 ng/L), people carrying C allele had a higher risk of liver damage. Although MC - LR enters into cells through SLCO1B1, no statistical differences between cases and controls were found in SLCO1B1 (T521C) with MC-LR exposure on liver damage whether at a low or a high MC-LR exposure level.

**Table 3:** Genotype and allele frequencies of SLCO1B1 (T521C) when participants were exposed to AFB1 or MC-LR in cases and controls.

Toxin Exposure	SNP	Cases n(%)	Controls n(%)	OR (95%CI)	P-value^a,b^
AFB1(L)	TT	158 (70.5)	147 (62.3)	ref	
	TC/CC	66 (29.5)	89 (37.7)	0.690 (0.467–1.019)	0.038
	T	176 (39.3)	173 (35.3)	ref	
	C	272 (60.7)	317 (64.7)	0.843 (0.647–1.099)	0.117
AFB1(H)	TT	125 (56.1)	171 (76.7)	ref	
	TC/CC	98 (43.9)	52 (23.3)	2.578 (1.715–3.876)	0.000
	T	142 (31.8)	186 (42.5)	ref	
	C	304(68.2)	252(57.2)	1.580 (1.200–2.080)	0.001
MC-LR(L)	TT	140 (62.2)	160 (67.5)	ref	
	TC/CC	85 (37.8)	77 (32.5)	1.262 (0.860–1.850)	0.137
	T	155 (34.4)	168 (36.1)	ref	
	C	295 (65.6)	298 (63.9)	1.073 (0.818–1.407)	0.330
MC-LR(H)	TT	143 (64.4)	158 (71.2)	ref	
	TC/CC	79 (35.6)	64 (28.8)	1.364 (0.914–2.034)	0.077
	T	163 (36.7)	191 (41.3)	ref	
	C	281 (63.3)	271 (58.7)	1.215 (0.930–1.588)	0.087

Note: ^a^Comparing Cases with Controls. ^b^From chi-square test.

L stood for low exposure, and H stood for high exposure.

### Interaction of SLCO1B1 (T521C) with AFB1 and MC-LR co-exposure on liver damage

Table 4 shows the joint effects of SLCO1B1 (T521C) with AFB1 and MC-LR co-exposure on human liver damage. There was no combined relationship between SLCO1B1 (T521C) with low AFB1 and low MC-LR co-exposure and liver damage, and it was the same with low AFB1 and high MC-LR co-exposure. For high AFB1 and low MC-LR co-exposure, the OR of liver damage was 2.838 in individuals carrying the mutation genotypes TC and CC *versus* those carrying the wild genotype TT (95%CI: 1.598-5.041, *P* < 0.05), and the frequencies of mutation genotypes (TC and CC) were 47.4% in cases and 24.1% in controls; the OR of liver damage was 1.632 in individuals carrying the mutation allele C *versus* those carrying the wild allele T (95%CI: 1.094-2.435, *P* < 0.05), and the frequencies of mutation allele C were 71.5% in cases and 60.6% in controls.

**Table 4:** Genotype and allele frequencies of SLCO1B1 (T521C) when participants were co-exposed to AFB1 and MC-LR in cases and controls.

Toxin Exposure	SNP	Cases n(%)	Controls n(%)	OR (95%CI)	P-value^a,b^
A_L_M_L_	TT	80 (72.1)	78 (60.5)	ref	
	TC/CC	31 (27.9)	51 (39.5)	0.593 (0.344–1.022)	0.039
	T	90 (40.5)	86 (33.3)	ref	
	C	132 (59.5)	172 (66.7)	0.733 (0.505–1.064)	0.062
A_L_M_H_	TT	78 (69.0)	69 (64.5)	ref	
	TC/CC	35 (31.0)	38 (35.5)	0.815 (0.465–1.429)	0.280
	T	86 (38.1)	87 (37.5)	ref	
	C	140 (61.9)	145 (62.5)	0.977 (0.669–1.425)	0.490
A_H_M_L_	TT	60 (52.6)	82 (75.9)	ref	
	TC/CC	54 (47.4)	26 (24.1)	2.838 (1.598–5.041)	0.000
	T	65 (28.5)	82 (39.4)	ref	
	C	163 (71.5)	126 (60.6)	1.632 (1.094–2.435)	0.011
A_H_M_H_	TT	65 (59.6)	89 (77.4)	ref	
	TC/CC	44 (40.4)	26 (22.6)	2.317 (1.296–4.142)	0.003
	T	77 (35.3)	104 (45.2)	ref	
	C	141 (64.7)	126 (54.8)	1.511 (1.033–2.211)	0.021

Note: ^a^Comparing Cases with Control. ^b^From chi-square test.

AM meant combined exposure to AFB1 and Microcystin-LR. A_L_M_L_ was defined as those with AFB1 lower than median and with MC-LR lower than median, and so on.

For high AFB1 and high MC-LR co-exposure, the risk value of liver damage in individuals with genotype SLCO1B1 (T521C)-TC/CC was 2.317 (95%CI: 1.296-4.142, *P* < 0.05) compared with those carrying wild genotype SLCO1B1(T521C)-TT, and the frequencies of mutation genotypes (TC and CC) were 40.4% in cases and 22.6% in controls; the OR was 1.511 in individuals carrying the mutation allele C *versus* those carrying the wild allele T (95%CI: 1.033-2.211, *P* < 0.05), and the frequencies of mutation allele C were 64.7% and 54.8% in cases and controls, respectively. These results showed that people carrying mutation allele C for SLCO1B1 (T521C) had a higher liver damage risk when exposed to a high AFB1 level no matter with a low or a high MC-LR level.

### Interaction of GSTP1 (A1578G) with AFB1 or MC-LR on liver damage

For AFB1 exposure, we found no interaction between GSTP1 (A1578G) with a low or high AFB1 level and human liver damage (Table 5). While for low MC-LR exposure, there were significant differences between mutation genotypes GSTP1 (A1578G)-AG/GG and wild genotypes GSTP1 (A1578G)-AA. The OR for GSTP1(A1578G) genotype AG/GG *versus* GSTP1 (A1578G) genotype AA was 0.659 (95%CI: 0.458-0.947, *P* < 0.05), and the frequencies of GSTP1 (A1578G)-AG/GG were 33.3% in cases and 43.2% in controls; for mutation allele G, OR was 0.657 (95%CI: 0.507-0.852, *P* < 0.05) compared with those carrying wild allele A, and the frequencies of G were 31.6% and 41.2% in cases and controls, respectively. Possibly, when exposed to a low MC-LR level, individuals carrying mutation genotype or allele of GSTP1 (A1578G) might have a lower risk of liver damage. When exposed to a high MC-LR level, individuals carrying GSTP1 (A1578G) mutation allele G might have a higher risk of liver damage than those carrying wild allele A [OR = 1.458, (95%CI: 1.069-1.987), *P* < 0.05], and the frequencies of G were 27.7% and 20.8% in cases and controls, respectively. However, we did not find the same trend for genotypes of GSTP1 (A1578G).

**Table 5:** Genotype and allele frequencies of GSTP1 (A1578G) when participants were exposed to AFB1 or MC-LR in cases and controls.

Toxin Exposure	SNP	Cases n(%)	Controls n(%)	OR (95%CI)	P-value^a,b^
AFB1(L)	AA	169 (75.4)	172 (73.2)	ref	
	AG/GG	55 (24.6)	63 (26.8)	0.889 (0.584–1.351)	0.328
	A	344 (76.8)	352 (74.9)	ref	
	G	104 (23.2)	118 (25.1)	0.902 (0.666–1.221)	0.277
AFB1(H)	AA	136 (61.0)	161 (59.4)	ref	
	AG/GG	87 (39.0)	110 (40.6)	0.936 (0.652–1.345)	0.396
	A	285 (63.9)	333 (61.4)	ref	
	G	161 (36.1)	209 (38.6)	0.900 (0.694–1.167)	0.233
MC-LR(L)	AA	150 (66.7)	162 (56.8)	ref	
	AG/GG	75 (33.3)	123 (43.2)	0.659 (0.458–0.947)	0.015
	A	308 (68.4)	335 (58.8)	ref	
	G	142 (31.6)	235 (41.2)	0.657 (0.507–0.852)	0.001
MC-LR(H)	AA	155 (69.8)	171 (77.4)	ref	
	AG/GG	67 (30.2)	50 (22.6)	1.478 (0.966–2.263)	0.045
	A	321 (72.3)	350 (79.2)	ref	
	G	123 (27.7)	92 (20.8)	1.458 (1.069–1.987)	0.010

Note: ^a^Comparing Cases with Control. ^b^From chi-square test.

L stood for low exposure, and H stood for high exposure.

### Interaction of GSTP1 (A1578G) with AFB1 and MC-LR co-exposure on liver damage

For genotype and allele of GSTP1 (A1578G), we did not observe any significant relationships of SNP on human liver damage at a low AFB1 with low or high MC-LR co-exposure level (Table 6). When people were co-exposed to a high AFB1 and low MC-LR level, the OR of liver damage was 0.617 (95%CI: 0.436-0.873, *P* < 0.05) for mutation allele G *versus* wild allele A, and the frequencies of G were 38.2% and 50.0% in cases and controls, respectively. No such a trend was found in genotypes of GSTP1 exposed to the same toxins. But people carrying mutation allele G and with a high AFB1 and high MC-LR exposure level had a higher risk of liver damage than those carrying wild allele A [OR = 1.739, (95%CI: 1.146-2.641), *P* < 0.05], and the frequencies of G were 33.9% in cases and 22.8% in controls. There was no statistical difference between genotype GSTP1 (A1578G) and human liver damage under the same exposure condition. We did not find the interaction of SLCO1B1 (T521C) and GSTP1 (A1578G) with AFB1 and/or MC-LR on liver damage (data not shown).

**Table 6:** Genotype and allele frequencies of GSTP1 (A1578G) when participants were co-exposed to AFB1 and MC-LR in cases and controls.

Toxin Exposure	SNP	Cases n(%)	Controls n(%)	OR (95%CI)	P-value^a,b^
A_L_M_L_	AA	83 (74.8)	87 (68.0)	ref	
	AG/GG	28 (25.2)	41 (32.0)	0.716 (0.406–1.262)	0.155
	A	167 (75.2)	178 (69.5)	ref	
	G	55 (24.8)	78 (30.5)	0.752 (0.501–1.126)	0.100
A_L_M_H_	AA	86 (76.1)	85 (79.4)	ref	
	AG/GG	27 (23.9)	22 (20.6)	1.213 (0.641–2.295)	0.333
	A	177 (78.3)	174 (81.3)	ref	
	G	49 (21.7)	40 (18.7)	1.204 (0.755–1.921)	0.254
A_H_M_L_	AA	67 (58.8)	75 (47.8)	ref	
	AG/GG	47 (41.2)	82 (52.2)	0.642 (0.394–1.044)	0.048
	A	141 (61.8)	157 (50.0)	ref	
	G	87 (38.2)	157 (50.0)	0.617 (0.436–0.873)	0.004
A_H_M_H_	AA	69 (63.3)	86 (75.4)	ref	
	AG/GG	40 (36.7)	28 (24.6)	1.781 (0.999–3.172)	0.034
	A	144 (66.1)	176 (77.2)	ref	
	G	74 (33.9)	52 (22.8)	1.739 (1.146–2.641)	0.006

Note: ^a^Comparing Cases with Control. ^b^From chi-square test.

AM means combined exposure to AFB1 and Microcystin-LR. A_L_M_L_ was defined as those with AFB1 lower than median and with MC-LR lower than median, and so on.

## Discussion

The only route for human to be exposed to AFB1 is via contaminated food. Food is widespread contaminated with AFB1 in China. A study on AFB1 in corn from the high-incidence area for human hepatocellular carcinoma found that AFB1 level in 76% of the corns consumed by people in Guangxi exceeded the Chinese regulation value of 20 microg/kg^29^. In our study, we found that the serum levels of AFB1 were 3.132 ng/L in cases and 2.935 ng/L in controls.

Microcystins are a threat to animals and humans. Human can be exposed to MCs through several routes: the oral one is the most important by far, occurring by ingestion of contaminated drinking water or food (including dietary supplements) or water during recreational activities. In addition, dermal/inhalation exposure may be associated with the domestic use of water (i.e., during a shower) or with professional and recreational activities (i.e., fishing)^30^. We performed a cross-sectional investigation of chronic exposure to MC-LR in relation to childhood liver damage in Fuling study field (2009), finding that the average levels of serum MC-LR are 1.3 and 0.4 μg MC-LR eq/L in the high-exposed and the low-exposed children, respectively. However, in our study, the mean concentrations of serum MC-LR in cases and controls were 0.282 and 0.268 ng/L, respectively (2014). MC-LR decreased through five years of risk management on water environment.

The toxicity of AFB1 and MC-LR depends on the balance between accumulation and metabolism of the toxins. Although the transport and metabolism of AFB1 and MC-LR involve many genes, but in the eight SNPs we chose, only SLCO1B1 (T521C) and GSTP1 (A1578G) could modify the risk of liver damage among individuals exposed to different levels of AFB1 and/or MC-LR. We did not find any associations of other selected SNPs with AFB1 and/or MC-LR on liver damage, including SLCO1B3 (T334G), GSTT1 (−/+), GSTM1 (−/+), GSTA1 (C69T), CYP2E1 (C1019T) and CYP3A4 (A13871G).

OATPs are members of the solute carrier transporters (SLC) superfamily and are classified as the solute carriers of the OATPs (*SLCO*) gene family^31^. OATPs can be either tissue-specific or be expressed in multiple tissues throughout the body, and are responsible for the uptake of a wide range of substrates of the Na^+^-independent^32^. Therefore, OATPs are considered to be the uptake transporter, and typically transport secondary and tertiary chemicals through biological membranes.

The SNPs of OATPs (*SLCO*) gene family are associated with the pharmacokinetics of drugs and substrates. OATP1B1 and OATP1B3 are primarily expressed in human liver^33,34^. SLCO1B1 (T521C) is allele change of amino acid change (V174A), which reduces the function of OATP1B1 *in vivo* not only at the plasma membrane but also in the intracellular space^35^.

At present, there is no relationship between AFB1 and *SLCO* family, but in our study, we found a harmful effect of SLCO1B1 on human liver damage among populations with a high AFB1 level alone or combined with MC-LR exposure. That is to say, when people were exposed to high AFB1, those carrying SLCO1B1 (T521C) mutation allele may have a higher liver damage risk, no matter at a low or a high level of MC-LR. Under high AFB1 exposure, the OR of high MC-LR exposure is lower than that of low MC-LR exposure in genotype and base (2.317 *versus* 2.838, 1.511 *versus* 1.632). A low level of MC-LR may increase the uptake of AFB1 to elevate the risk of liver injury. However, no scientific research on the biological relationship between AFB1 and SLCO1B1 has been found. This requires in-depth studies to explain the mechanism of AFB1 and SLCO1B1.

Although the uptake of MC-LR into hepatocytes has been confirmed to be transported mainly through OATP1B1 and OATP1B3^36–39^, we did not find any differences in association between OATP1B1 (T521C) or SLCO1B3 (T334G) and MC-LR exposure alone or combined with AFB1 on risk of liver injury.

The P450 2E1 (CYP2E1) gene, located on chromosome10q26.3, spanning approximately 11.8 kb in length and consisting of 9 exons, is responsible for encoding a member enzyme of the cytochrome P450 superfamily involved in drug metabolism and is suggested to be associated with the risk of liver cancer^40^. The RsaI/PstI polymorphism in the promoter region of CYP2E1 (C1019T) gene has been reported to affect the transcriptional activity of CYP2E1^41^. AFB1 can stimulate the expression of CYP genes (only CYP1A1, CYP1B1, CYP3A4, CYP3A5 and CYP3A7) in monocytes more intensively than in lymphocytes^42^, and AFB1 is metabolized by cytochrome P450 3A4 (CYP3A4)^43,44^. However, we did not find effects of the interaction between polymorphism of CYP3A4 (A13871G) and AFB1 exposure on human liver damage. MC-LR could bioactivate CYP2E1 to produce ROS and free radicals. A low dose of MC-LR can induce the generation of ROS, and upregulate the expression of CYP2E1 mRNA, suggesting that CYP2E1 may be a potential factor responsible for ROS generation by MC-LR^45^. We found CYP2E1 (C1019T) polymorphism was not a susceptible factor for liver damage in persons exposed to MC-LR.

GSTs are phase II enzymes that can catalyze the conjugation of glutathione (GSH) to a wide variety of endogenous and exogenous electrophilic compounds. GSTs represent the multiple gene family of dimeric enzymes, and are distributed ubiquitously. In humans, polymorphism in GST genes has been associated with susceptibility to various diseases based on recent data, which indicates that these genotypes can modify disease phenotypes. Thus, GST genotypes alone and in combination with MC-LR exposure are linked with clinical outcomes^46^.

A study conducted in Guangxi, China^47^ found that GSTT1-null genotype increases the HCC risk, but another study did not find any significant associations. However, another study of China suggested that there is evidence showing the interaction of GSTM1 polymorphism with AFB1 exposure, particularly with low/median levels of AFB1 exposure [adjusted OR (95% CI) = 1.92 (0.92-4.00) and 1.80 (0.77–4.17)]. Individuals carrying GSTM1-null have an increased risk of developing HCC [adjusted OR (95% CI) = 2.07 (1.20–3.57)]^48^. GSTM1 deletion cannot effectively code the GSTM1 detoxification enzyme, then the GSTM1 detoxification enzyme of phase II enzymes cannot catalyze the conjugation of glutathione (GSH) and with AFB1. Therefore, AFB1 accumulates in the liver to exert toxicity and causes liver damage. The conjugation of glutathione to AFB1 by GSTs is a major pathway of detoxification^49^. AFB1 at a low activity dose could protect human hepatocytes against aflatoxin-DNA adduct formation in those with GSTM1-null^50^. In our preliminary findings, GSTT1 null genotype may be a susceptible factor for liver damage in persons exposed to MC-LR, There was no statistically significance in the distribution of GSTM1 genotypes in the two groups (*P* = 1, χ^2^ test). However, the frequency of GSTT1(−) in the case group was 60.5%, significantly higher than that in the control group (39.1%), and the difference was statistically significant (*P* = 0.006). In the case group, the joint frequency of GSTM1(−)/GSTT1(−) was 29.6%, higher than that in the control group (16.3%), and the risk of hepatic injury among individuals with GSTM1(−)/GSTT1(−) was 3.01 times and 2.54 times higher than that among those with GSTM1( + )/GSTT1( + ) and GSTM1(−)/GSTT1( + ), respectively. Hence, GSTM1 and GSTT1 null genotypes may increase the risk of liver damage in persons exposed to MC-LR^51^; however in our study, we did not find any effects of GSTT1 null genotype and GSTM1 null genotype with AFB1 or MC-LR on liver damage, which may be attributed to the sample size of our preliminary study.

Although we did not find any combinative effects of GSTA1 (C69T) with AFB1 and/or MC-LR exposure on human liver damage, various studies have shown marked inter-individual variation in expression of GSTA1 in human tissues^52^. GSTA1 is related to AFB1 or MC-LR. In salmonella typhimurium tester strains, they find a pronounced reduction role of GSTA1 in AFB1 deactivation^53^. GSTA1 seems to be mainly involved in the MC-LR detoxification in liver after oral exposure^30^.

A functional sequence variant in GSTP1 at codon 105 has been associated with many types of tumors. GSTP1 could significantly increase the conjugation of AFBO with glutathione^54^; a statistically significant association is found between GSTP1 promoter hypermethylation and the level of AFB1-DNA adducts in tumor tissues (OR = 2.81, 95% CI: 1.03-7.70)^55^. GSTP1 kinetics and location in the skin suggest some effects related to dermal exposure to MC-LR^30^. There were no significant differences between GSTP1 (A1578G) and liver damage among individuals with a low level of AFB1 exposure alone or in combination with MC-LR or among those with a high level of AFB1 exposure alone. However, we found that GSTP1 (A1578G) might have harmful effects on the liver when interacted with a high MC-LR level alone or combined with a high AFB1 level, and that GSTP1 might have protective effects when interacted with a low MC-LR level alone or combined with a high AFB1 level, which may be beyond the functional change of amino acids (Ile 105 Val). We speculate that at low MC-LR exposure levels, MC-LR may be absorbed mainly through the skin, during which process GSTP1 (A1578G) is a protective gene, and that at high MC-LR exposure levels, other GSTs, not including GSTP1, is a major metabolic factor.

There are limitations to this study. First, we did not examine the polymorphisms of all transport and metabolic genes associated with AFB1 and MC-LR; second, our study lacks further functional verification in joint effects between genes and toxins; third, we did not find the relationship between OATPB1 and AFB1.

In conclusion, the related transport and metabolic genes of AFB1 and MC-LR, namely SLCO1B1 (T521C) and GSTP1 (A1578G), are susceptible genes of liver damage among populations exposed to AFB1 alone or combined with MC-LR. Therefore, in our risk management of food and water, we should pay special attention to people carrying mutation genotypes of SLCO1B1 (T521C) or GSTP1 (A1578G).

## Material and Methods

### Ethics statement

This study was approved by the Ethics Committee of the Third Military Medical University. All methods were performed in accordance with the relevant guidelines and regulations of Third Military Medical University. Informed consent was obtained from all subjects.

### Study population and data collection

This study is a cross-sectional investigation on relationship of joint exposure to AFB1 and MC-LR at low levels with liver damage in Fuling district of Chongqing, China, in July 2013. Briefly, we randomly recruited study populations from two towns (n = 6467) of Fuling, administered questionnaires about demographic information, the history of disease, the risk factors for liver damage (including information of drinking water and diet) of each participant, and then collected their blood samples to measure liver function indicators and serum AFB1 and MC-LR levels using ELISA assay. We excluded participants with HBV or HCV infection or using medicines with liver toxicity. Cases were participants with liver damage defined as with at least one abnormal serum enzyme detected, and controls were those without any clinical liver damage and randomly selected from the cross-sectional investigation. To control the effects of confounders, controls were individually matched to cases (1:1) based on sex, ethnicity, age (± 5years), and residence. Finally, there were 475 cases and 475 controls enlisted, among which the interaction of the eight SNPs with exposure to MC-LR and AFB1 on liver damage was observed. At the same time, 5 mL of peripheral blood was obtained for serum analysis and DNA extraction.

### DNA extraction and genotyping

The genomic DNA was extracted from the whole blood using Wizard Genomic Purification Kit (Cat^.#^A1120Promega Corporation, USA). DNA samples were stored at −80 °C until processed. Polymorphisms of SLCO1B1 (T521C), SLCO1B3 (T334G), GSTA1 (C69T), GSTP1 (A1578G), CYP2E1 (C1019T) and CYP3A4 (A13871G) were genotyped by restriction fragment length polymorphism (RFLP) analysis. The deletion alleles of GSTM1 and GSTT1 were analyzed by a polymerase chain reaction (PCR) approach. As a positive control, co-amplification of the 268-bp fragment of the β-globin gene was performed at the same time as the analysis of GSTM1 and GSTT1 polymorphisms. Primer sequences of eight SNPs and β-globin, restriction enzymes of six SNPs, and diagnostic DNA fragments are listed in Table 7.

**Table 7:** PCR primers, restriction enzymes and diagnostic DNA fragments used in the experiment.

No.	Target gene	Primer sequence (5′-3′)		DNA fragment (bp)	Restriction enzyme
		Forward	Reverse		
1	GSTM1 (−/+)^a^	CTCCTGATTATGACAGAAGCC	CTGGATTGTAGCAGATCATGG	650	—
2	GSTT1 (−/+)^a^	TTCCTTACTGGTCCTCACATCTC	TCACCGGATCATGGCCAGCA	480	—
3	β-Globulin^a^	CAACTTCATCCACGTTCACC	GAAGAGCCAAGGACAGGTAC	268	—
4	GSTA1 (*C69T*)^b^	TGTTGATTGTTTGCCTGAAATT	GTTAAACGCTGTCACCCGTCCT	480 (380/100)	EarI
5	GSTP1 (*A1578G*)^b^	ACCCCAGGGCTCTATGGGAA	TGAGGGCACAAGAAGCCCCT	176 (91/85)	BsmAI
6	CYP2E1 (*C1019T*)^b^	TTCATTCTGTCTTCTAACTGG	CCAGTCGAGTCTACATTGTCA	410 (360/50)	RsaI
7	CYP3A4 (*A13871G*)^b^	CACATTTTCTACAACCATGGAGACC	GAATCTGGTGGGGACAGGTATAAA	249 (14/141/94)	BsmAI
8	SLCO1B1 (*T521C*)^b^	TTGTCAAAGTTTGCAA AGTG	GAAGCATATTACCCATGAGC	209 (189/20)	Hha I
9	SLCO1B3 (*T334G*)^b^	GAAGGTACAATGTCTTGGGC	CTCTCAAAAGGTAACTGCC	253 (213/40)	Alu I

Note: ^a^analyzed by PCR approach. ^b^genotyped by RFLP analysis.

Genotyping was performed by laboratory personnel blinded to case-control status, and a random 5% of the samples were repeated to validate genotyping procedure with identical results.

### Serum liver enzyme analysis

Serum was isolated, and alanine aminotransferase (ALT) and aspartate aminotransferase (AST) were assayed using Synchron Clinical System LX20 (Beckman-Coulter Diagnosis, Fullerton, CA, USA). Normal ranges of ALT and AST were defined as 7~40 U/L and 8~40 U/L, respectively.

### AFB1 in serum

AFB1-albumin adduct levels in serum were measured by enzyme-linked immunosorbent assay. Serum samples and AFB1-albumin adduct standards (10.0 ng/ml) were added to the plate. The monoclonal antibody solution of AFB1-albumin adduct was added into the wells and incubated for 30 minutes at 37 °C temperature. After washing five times, samples were added with color solutions A and B and incubated for 10 min at 37 °C temperature. Finally, read at absorbance of 450 nm using the ELISA photometer after adding stop solutions. The limit of detection (LOD) of this assay was 0.1 ng/ml, and percent recovery of this assay was >80.

### MC-LR in serum

The direct competitive enzyme-linked immunosorbent assay (dcELISA) was used to determine the level of MC-LR in serum. In the assay, MC toxin in the sample competes with horseradish peroxidase-conjugated MCs for a limited amount of antibody which has been coated on the bottom of the test wells. Briefly, we added 50 μL of MCY-HRP enzyme conjugate solution (HRP conjugate) to all wells after adding 50 μL of Negative control and standard solution (0.1, 0.5, 1.0, 2.0, and 5.0 ppb) or 50 μL of each sample into the assigned well. The plate was gently swirled to mix the content thoroughly. After incubation at room temperature (25–37 °C) for 30 minutes in dark, we removed the liquid from all wells which were then flooded with at least 300–350 μL of 1 x washing buffer, and from which the liquid was decanted. The washing step was repeated at least three times. The plate was then inverted and gently patted on absorbent paper towels to remove remaining solution in wells followed by the step of adding 100 μL of substrate solution to each well and shaking gently. After incubation for 30 minutes at room temperature (25–37 °C) in dark, blue color developed in the wells with Negative control. The solution then turned from blue into yellow immediately after adding 100 μL of stop solution to each well and mixing gently. Read color at OD 450 nm in an ELISA reader within 3–15 minutes after adding the stop solution. The detection limit for this assay based on MC-LR is 0.01 ppb (ng/mL).

In this study, serum specimens were grouped into case-control pairs and were assayed on the same day to minimize any effects of day-to-day laboratory variation.

### Different exposure levels of MC-LR and AFB1

To find the effects of co-exposure to AFB1 and MC-LR on liver damage, we divided all the participants into four groups based on different exposure levels of the two toxins by median, and the medians of MC-LR and AFB1 were 0.21 and 2.93 ng/L, respectively. The four groups were A_L_M_L,_ A_L_M_H_, A_H_M_L_ and A_H_M_H_. A_L_M_L_ meant the group with a low exposure level of AFB1 co-exposed to a low level of MC-LR, and so on. Among them, A is the abbreviation of AFB1, M of MC-LR, L of a low exposure level of the toxin, and H of a high exposure level of the toxin. Accordingly, MC-LR (H) meant the group with a high exposure level of MC-LR, and so on.

### Statistical analysis

We tested deviation from Hardy-Weinberg equilibrium using chi-square analysis. The *t* test was used to examine the differences in sex, age, BMI, daily water intake, residence, income, ALT, AST, serum AFB1 level and serum MC-LR level between cases and controls. Alcohol drinking status, smoking, passive smoking, tea drinking habit between cases and controls were analyzed by Chi-square test. Chi-square test was used to examine differences in the distributions and frequencies of mutation genotypes and alleles of the eight SNPs between cases and controls adjusted for BMI. Associations between genotypes (classficative variables) and liver damage (quantificative variable) were analyzed with one-way analysis of variance (ANOVA). Statistical analysis was conducted using the IBM SPSS Statistical 20.0 (StaSoft Enc., Tulsa, OK, USA). All *P* values quoted are one-sided. One-sided *P* values that are 0.05 or less were considered statistically significant.

## Electronic supplementary material

Supplementary material
