# Supplementary material for: Interaction Effects of AFB1 and MC-LR Co-exposure with Polymorphism of Metabolic Genes on Liver Damage: focusing on SLCO1B1 and GSTP1
Source: Sci Rep. 2017 Nov 23;7:16164. doi: 10.1038/s41598-017-16432-z (PMC5700940; doi:10.1038/s41598-017-16432-z)
Supplement: Supplementary file 1 — Supplementary material [file 41598_2017_16432_MOESM1_ESM.doc]

**Title:** **Interaction Effects of AFB1 and MC-LR Co-exposure with Polymorphism of Metabolic Genes on Liver Damage: focusing on SLCO1B1 and GSTP1**

Xiaohong Yang^1^,Wenyi Liu^1^, Hui Lin,^2^ Hui Zeng^1^, Renping Zhang^3^, Chaowen Pu^3^, Lingqiao Wang^1^, Chuanfen Zheng^1^, Yao Tan^1^, Yang Luo^4^, Xiaobin Feng^5^, Yingqiao Tian^3^, Guosheng Xiao^6^, Jia Wang^1^, Yujing Huang^1^, Jiaohua Luo^1^, Zhiqun Qiu^1^, Ji-an Chen^7^, Liping Wu^1^, Lixiong He^1^ and Weiqun Shu^1^*

^1^Department of Environmental Hygiene, College of Preventive Medicine, Third Military Medical University, Chongqing, 400038, China; ^2^Department of Tropical Epidemiology, College of Preventive Medicine, Third Military Medical University, Chongqing, 400038, China; ^3^The Center for Disease Control and Prevention in Fuling District, Chongqing, 408000, China; ^4^Center for Nanomedicine, Southwest Hospital, Third Military Medical University, Chongqing, 400038, China; ^5^Institute of Hepatobiliary Surgery, Southwest Hospital, Third Military Medical University, Chongqing, 400038, China; ^6^College of Life Science and Engineering, Chongqing Three Gorges University, Wanzhou, Chongqing, 404100, China; ^7^Department of Health Education, College of Preventive Medicine, Third Military Medical University, Chongqing, 400038, China.

**Address correspondence to** Weiqun Shu*. Department of Environmental Hygiene, College of Preventive Medicine, Third Military Medical University, Chongqing, China. Telephone: 86-23-68772309. E-mail: xm0630@sina.com.

**Running title:** SLCO1BI (T521C) and GSTP1 (A1578G) altered liver damage among AFB1 and MC-LR exposed populations.

**Table S1.** Genotype and allele frequencies of SLCO1B3 (T334G) when participants were exposed to AFB1 or MC-LR in cases and controls

| **Toxin Exposure** | **SLCO1B3** | **Cases n(%)** | **Controls n(%)** | **OR（95%CI）** | **P-value**^a,b^ |
| --- | --- | --- | --- | --- | --- |
| MC-LR(L) | TT | 144(64.86) | 151(65.37) | ref. |  |
|  | TG/GG | 78(35.14) | 80(34.63) | 1.022(0.695-1.505) | 0.494 |
|  | T | 158(35.59) | 170(36.8) | ref. |  |
|  | G | 286(64.41) | 292(63.2) | 1.054(0.804-1.382) | 0.378 |
| MC-LR(H) | TT | 133(61.86) | 142(66.05) | ref. |  |
|  | TG/GG | 82(38.14) | 73(33.95) | 1.199(0.809-1.779) | 0.211 |
|  | T | 152(35.35) | 167(38.84) | ref. |  |
|  | G | 278(64.65) | 263(61.16) | 1.161(0.880-1.532) | 0.162 |
| AFB1(L) | TT | 132(61.11) | 154(67.25) | ref. |  |
|  | TG/GG | 84(38.89) | 75(32.75) | 1.307(0.886-1.927) | 0.105 |
|  | T | 148(34.26) | 171(37.34) | ref. |  |
|  | G | 284(65.74) | 287(62.66) | 1.143(0.869-1.504) | 0.188 |
| AFB1(H) | TT | 145(65.61) | 139(64.06) | ref. |  |
|  | TG/GG | 76(34.39) | 78(35.94) | 0.934(0.631-1.383) | 0.405 |
|  | T | 162(36.65) | 166(38.25) | ref. |  |
|  | G | 280(63.35) | 268(61.75) | 1.071(0.814-1.408) | 0.338 |

Note: ^a^Comparing Cases with Controls. ^b^From chi-square test, adjusted for BMI.

L stood for low exposure, and H stood for high exposure.

**Table S2.** Genotype and allele frequencies of SLCO1B3 (T334G) when participants were co-exposed to AFB1 and MC-LR in cases and controls

| **Toxin Exposure** | **SLCO1B3** | **Cases n(%)** | **Controls n(%)** | **OR（95%CI）** | **P-value**^a,b^ |
| --- | --- | --- | --- | --- | --- |
| A_L_M_L_ | TT | 67(62.04) | 88(69.84) | ref. |  |
|  | TG/GG | 41(37.96) | 38(30.16) | 1.417(0.823-2.441) | 0.131 |
|  | T | 75(34.72) | 93(36.9) | ref. |  |
|  | G | 141(65.28) | 159(63.1) | 1.100(0.752-1.607) | 0.347 |
| A_L_M_H_ | TT | 65(60.19) | 66(64.08) | ref. |  |
|  | TG/GG | 43(39.81) | 37(35.92) | 1.180(0.676-2.060) | 0.33 |
|  | T | 73(33.8) | 78(37.86) | ref. |  |
|  | G | 143(66.2) | 128(62.14) | 1.194(0.801-1.778) | 0.221 |
| A_H_M_L_ | TT | 77(67.54) | 63(60) | ref. |  |
|  | TG/GG | 37(32.46) | 42(40) | 0.721(0.414-1.253) | 0.154 |
|  | T | 83(36.4) | 77(36.67) | ref. |  |
|  | G | 145(63.6) | 133(63.33) | 1.011(0.685-1.493） | 0.517 |
| A_H_M_H_ | TT | 68(63.55) | 76(67.86) | ref. |  |
|  | TG/GG | 39(36.45) | 36(32.14) | 1.211(0.692-2.117) | 0.298 |
|  | T | 79(36.92) | 89(39.73) | ref. |  |
|  | G | 135(63.08) | 135(60.27) | 1.127(0.766-1.657) | 0.306 |

Note: ^a^Comparing Cases with Controls. ^b^From chi-square test, adjusted for BMI.

A+M meant combined exposure to AFB1 and microcystin-LR. A_L_M_L_ was defined as those with AFB1 lower than median and with MC-LR lower than median, and so on.

**Table S3.** Genotype and allele frequencies of GSTT1(+/-) and GSTM1(+/-) when participants were exposed to AFB1 or MC-LR in cases and controls

| **Toxin Exposure** | **Gene** |  | **Cases n(%)** | **Controls n(%)** | **OR（95%CI）** | **P-value**^a,b^ |
| --- | --- | --- | --- | --- | --- | --- |
| MC-LR(L) | GSTT1 | + | 116(51.56) | 119(50.64) | ref. |  |
|  |  | - | 109(48.44) | 116(49.36) | 0.964(0.669-1.390) | 0.459 |
| MC-LR(H) | GSTT1 | + | 104(46.85) | 116(52.02) | ref. |  |
|  |  | - | 118(53.15) | 107(47.98) | 1.230(0.848-1.785) | 0.16 |
| AFB1(L) | GSTT1 | + | 117(52.23) | 123(52.12) | ref. |  |
|  |  | - | 107(47.77) | 113(47.88) | 0.995(0.690-1.435) | 0.528 |
| AFB1(H) | GSTT1 | + | 103(46.19) | 112(50.45) | ref. |  |
|  |  | - | 120(53.81) | 110(49.55) | 1.186(0.818-1.721) | 0.211 |
| MC-LR(L) | GSTM1 | + | 90(68.44) | 103(58.77) | ref. |  |
|  |  | - | 135(31.56) | 135(41.23) | 1.144(0.791-1.657) | 0.267 |
| MC-LR(H) | GSTM1 | + | 99(72.3) | 80(79.19) | ref. |  |
|  |  | - | 132(27.7) | 144(20.81) | 0.741(0.508-1.081) | 0.072 |
| AFB1(L) | GSTM1 | + | 81(76.79) | 90(74.89) | ref. |  |
|  |  | - | 143(23.21) | 148(25.11) | 1.074(0.736-1.567) | 0.393 |
| AFB1(H) | GSTM1 | + | 108(63.9) | 93(61.44) | ref. |  |
|  |  | - | 124(36.1) | 131(38.56) | 0.815(0.563-1.180) | 0.162 |

Note: ^a^Comparing Cases with Controls. ^b^From chi-square test, adjusted for BMI.

L stood for low exposure, and H stood for high exposure.

**Table S4.** Genotype and allele frequencies of GSTT1(+/-) and GSTM1(+/-) when participants were co-exposed to AFB1 and MC-LR in cases and controls

| **Toxin Exposure** | **Gene** |  | **Cases n(%)** | **Controls n(%)** | **OR（95%CI）** | **P-value**^a,b^ |
| --- | --- | --- | --- | --- | --- | --- |
| A_L_M_L_ | GSTT1 | + | 60(54.05) | 65(51.18) | ref. |  |
|  |  | - | 51(45.95) | 62(48.82) | 0.891(0.535-1.484) | 0.377 |
| A_L_M_H_ | GSTT1 | + | 57(50.44) | 58(53.21) | ref. |  |
|  |  | - | 56(49.56) | 51(46.79) | 1.117(0.660-1.892) | 0.39 |
| A_H_M_L_ | GSTT1 | + | 56(49.12) | 54(50) | ref. |  |
|  |  | - | 58(50.88) | 54(50) | 1.036(0.612-1.753) | 0.501 |
| A_H_M_H_ | GSTT1 | + | 47(43.12) | 58(50.88) | ref. |  |
|  |  | - | 62(56.88) | 56(49.12) | 1.366(0.806-2.316) | 0.152 |
| A_L_M_L_ | GSTM1 | + | 41(75.23) | 60(69.53) | ref. |  |
|  |  | - | 70(24.77) | 69(30.47) | 1.485(0.884-2.492) | 0.833 |
| A_L_M_H_ | GSTM1 | + | 40(78.32) | 30(81.31) | ref. |  |
|  |  | - | 73(21.68) | 79(18.69) | 0.693(0.392-1.226) | 0.132 |
| A_H_M_L_ | GSTM1 | + | 49(61.84) | 43(50) | ref. |  |
|  |  | - | 65(38.16) | 66(50) | 0.864(0.507-1.474) | 0.345 |
| A_H_M_H_ | GSTM1 | + | 59(66.06) | 50(77.19) | ref. |  |
|  |  | - | 59(33.94) | 65(22.81) | 0.769(0.459-1.289) | 0.193 |

Note: ^a^Comparing Cases with Controls. ^b^From chi-square test, adjusted for BMI.

A+M meant combined exposure to AFB1 and microcystin-LR. A_L_M_L_ was defined as those with AFB1 lower than median and with MC-LR lower than median, and so on.

**Table S5.** Genotype and allele frequencies of GSTA1 (C69T) when participants were exposed to AFB1 or MC-LR in cases and controls

| **Toxin Exposure** | **GSTA1** | **Cases n(%)** | **Controls n(%)** | **OR（95%CI）** | **P-value**^a,b^ |
| --- | --- | --- | --- | --- | --- |
| MC-LR(L) | CC | 169(79.72) | 162(76.42) | ref. |  |
|  | CT/TT | 43(20.28) | 50(23.58) | 0.842(0.520-1.307) | 0.241 |
|  | C | 342(80.66) | 325(76.65) | ref. |  |
|  | T | 82(19.34) | 99(23.35) | 0.787(0.566-1.095) | 0.090 |
| MC-LR(H) | CC | 177(79.73) | 146(78.07) | ref. |  |
|  | CT/TT | 45(20.27) | 41(21.93) | 0.905(0.562-1.458) | 0.386 |
|  | C | 358(80.63) | 294(78.61) | ref. |  |
|  | T | 86(19.37) | 80(21.39) | 0.883(0.628-1.242) | 0.265 |
| AFB1(L) | CC | 179(79.91) | 190(80.51) | ref. |  |
|  | CT/TT | 45(20.09) | 46(19.49) | 1.038(0.656-1.643) | 0.482 |
|  | C | 364(81.25) | 380(80.51) | ref. |  |
|  | T | 84(18.75) | 92(19.49) | 0.953(0.666-1.324) | 0.42 |
| AFB1(H) | CC | 167(79.52) | 118(72.39) | ref. |  |
|  | CT/TT | 43(20.48) | 45(27.61) | 0.675(0.418-1.091) | 0.069 |
|  | C | 336(80) | 239(73.31) | ref. |  |
|  | T | 84(20) | 87(26.69) | 0.687(0.488-0.967) | 0.020 |

Note: ^a^Comparing Cases with Controls. ^b^From chi-square test, adjusted for BMI.

L stood for low exposure, and H stood for high exposure.

**Table S6.** Genotype and allele frequencies of GSTA1 (C69T) when participants were co-exposed to AFB1 and MC-LR in cases and controls

| **Toxin Exposure** | **GSTA1** | **Cases n(%)** | **Controls n(%)** | **OR（95%CI）** | **P-value**^a,b^ |
| --- | --- | --- | --- | --- | --- |
| A_L_M_L_ | CC | 93(83.78) | 104(81.89) | ref. |  |
|  | CT/TT | 18(16.22) | 23(18.11) | 0.875(0.445-1.723) | 0.416 |
|  | C | 188(84.68) | 208(81.89) | ref. |  |
|  | T | 34(15.32) | 46(18.11) | 0.818(0.503-1.328) | 0.245 |
| A_L_M_H_ | CC | 86(76.11) | 86(78.9) | ref. |  |
|  | CT/TT | 27(23.89) | 23(21.1) | 1.174(0.624-2.207) | 0.368 |
|  | C | 176(77.88) | 172(78.9) | ref. |  |
|  | T | 50(22.12) | 46(21.1) | 1.062(0.676-1.670) | 0.442 |
| A_H_M_L_ | CC | 76(75.25) | 58(68.24) | ref. |  |
|  | CT/TT | 25(24.75) | 27(31.76) | 0.707(0.372-1.343) | 0.185 |
|  | C | 154(76.24) | 117(68.82) | ref. |  |
|  | T | 48(23.76) | 53(31.18) | 0.688(0.435-1.088) | 0.069 |
| A_H_M_H_ | CC | 91(83.49) | 60(76.92) | ref. |  |
|  | CT/TT | 18(16.51) | 18(23.08) | 0.659(0.318-1.368) | 0.175 |
|  | C | 182(83.49) | 122(78.21) | ref. |  |
|  | T | 36(16.51) | 34(21.79) | 0.710(0.421-1.196) | 0.124 |

Note: ^a^Comparing Cases with Controls. ^b^From chi-square test, adjusted for BMI.

A+M meant combined exposure to AFB1 and microcystin-LR. A_L_M_L_ was defined as those with AFB1 lower than median and with MC-LR lower than median, and so on.

**Table S7.** Genotype and allele frequencies of CYP2E1 (C1019T) when participants were exposed to AFB1 or MC-LR in cases and controls

| **Toxin Exposure** | **CYP2E1** | **Cases n(%)** | **Controls n(%)** | **OR（95%CI）** | **P-value**^a,b^ |
| --- | --- | --- | --- | --- | --- |
| MC-LR(L) | CC/CT | 145(64.73) | 150(64.94) | ref. |  |
|  | TT | 79(35.27) | 81(35.06) | 1.009(0.687-1.483) | 0.521 |
|  | C | 155(34.6) | 159(34.42) | ref. |  |
|  | T | 293(65.4) | 303(65.58) | 0.992(0.755-1.304) | 0.505 |
| MC-LR(H) | CC/CT | 153(69.23) | 140(63.06) | ref. |  |
|  | TT | 68(30.77) | 82(36.94) | 0.759(0.511-1.126) | 0.102 |
|  | C | 159(35.97) | 148(33.33) | ref. |  |
|  | T | 283(64.03) | 296(66.67) | 0.890(0.675-1.174) | 0.225 |
| AFB1(L) | CC/CT | 156(70.27) | 152(64.96) | ref. |  |
|  | TT | 66(29.73) | 82(35.04) | 0.784(0.529-1.163) | 0.133 |
|  | C | 165(37.16) | 160(34.19) | ref. |  |
|  | T | 279(62.84) | 308(65.81) | 0.878(0.670-1.152) | 0.193 |
| AFB1(H) | CC/CT | 142(63.68) | 138(63.01) | ref. |  |
|  | TT | 81(36.32) | 81(36.99) | 0.972(0.660-1.431) | 0.482 |
|  | C | 149(33.41) | 147(33.56) | ref. |  |
|  | T | 297(66.59) | 291(66.44) | 1.007(0.761-1.331) | 0.509 |

Note: ^a^Comparing Cases with Controls. ^b^From chi-square test, adjusted for BMI.

L stood for low exposure, and H stood for high exposure.

**Table S8.** Genotype and allele frequencies of CYP2E1 (C1019T) when participants were co-exposed to AFB1 and MC-LR in cases and controls

| **Toxin Exposure** | **CYP2E1** | **Cases n(%)** | **Controls n(%)** | **OR（95%CI）** | **P-value**^a,b^ |
| --- | --- | --- | --- | --- | --- |
| A_L_M_L_ | CC/CT | 78(70.91) | 80(63.49) | ref. |  |
|  | TT | 32(29.09) | 46(36.51) | 0.713(0.412-1.235) | 0.142 |
|  | C | 84(38.18) | 84(33.33) | ref. |  |
|  | T | 136(61.82) | 168(66.67) | 0.810(0.555-1.181) | 0.158 |
| A_L_M_H_ | CC/CT | 78(69.64) | 72(66.67) | ref. |  |
|  | TT | 34(30.36) | 36(33.33) | 0.872(0.494-1.538) | 0.371 |
|  | C | 81(36.16) | 76(35.19) | ref. |  |
|  | T | 143(63.84) | 140(64.81) | 0.958(0.649-1.416) | 0.455 |
| A_H_M_L_ | CC/CT | 67(58.77) | 70(66.67) | ref. |  |
|  | TT | 47(41.23) | 35(33.33) | 1.403(0.809-2.434) | 0.143 |
|  | C | 71(31.14) | 75(35.71) | ref. |  |
|  | T | 157(68.86) | 135(64.29) | 1.228(0.825-1.829) | 0.181 |
| A_H_M_H_ | CC/CT | 75(68.81) | 68(59.65) | ref. |  |
|  | TT | 34(31.19) | 46(40.35) | 0.670(0.386-1.163) | 0.099 |
|  | C | 78(35.78) | 72(31.58) | ref. |  |
|  | T | 140(64.22) | 156(68.42) | 0.828(0.559-1.228) | 0.201 |

Note: ^a^Comparing Cases with Controls. ^b^From chi-square test, adjusted for BMI.

A+M meant combined exposure to AFB1 and microcystin-LR. A_L_M_L_ was defined as those with AFB1 lower than median and with MC-LR lower than median, and so on.

**Table S9.** Genotype and allele frequencies of CYP3A4 (A13781G) when participants were exposed to AFB1 or MC-LR in cases and controls

| **Toxin Exposure** | **CYP3A4** | **Cases n(%)** | **Controls n(%)** | **OR（95%CI）** | **P-value**^a,b^ |
| --- | --- | --- | --- | --- | --- |
| MC-LR(L) | AA | 155(68.89) | 173(73) | ref. |  |
|  | AG/GG | 70(31.11) | 64(27) | 1.221(0.816-1.825) | 0.192 |
|  | A | 313(29.7) | 321(29.56) | ref. |  |
|  | G | 741(70.3) | 765(70.44) | 0.993(0.825-1.196) | 0.491 |
| MC-LR(H) | AA | 164(73.87) | 171(76.34) | ref. |  |
|  | AG/GG | 58(26.13) | 53(23.66) | 1.141(0.743-1.753) | 0.311 |
|  | A | 295(28.92) | 312(29.66) | ref. |  |
|  | G | 725(71.08) | 740(70.34) | 1.036(0.858-1.252) | 0.375 |
| AFB1(L) | AA | 161(71.88) | 183(76.89) | ref. |  |
|  | AG/GG | 63(28.13) | 55(23.11) | 1.302(0.856-1.980) | 0.129 |
|  | A | 297(29.12) | 324(29.45) | ref. |  |
|  | G | 723(70.88) | 776(70.55) | 1.016(0.843-1.226) | 0.451 |
| AFB1(H) | AA | 158(70.85) | 161(72.2) | ref. |  |
|  | AG/GG | 65(29.15) | 62(27.8) | 1.068(0.708-1.612) | 0.417 |
|  | A | 311(29.51) | 309(29.77) | ref. |  |
|  | G | 743(70.49) | 729(70.23) | 1.013(0.839-1.222) | 0.467 |

Note: ^a^Comparing Cases with Controls. ^b^From chi-square test, adjusted for BMI.

L stood for low exposure, and H stood for high exposure.

**Table S10.** Genotype and allele frequencies of CYP3A4 (A13781G) when participants were co-exposed to AFB1 and MC-LR in cases and controls

| **Toxin Exposure** | **CYP3A4** | **Cases n(%)** | **Controls n(%)** | **OR（95%CI）** | **P-value**^a,b^ | |
| --- | --- | --- | --- | --- | --- | --- |
| A_L_M_L_ | AA | 71(63.96) | 103(79.84) | ref. | |  |
|  | AG/GG | 40(36.04) | 26(20.16) | 2.232(1.251-3.982) | | 0.005 |
|  | A | 148(29.37) | 176(29.53) | ref. | |  |
|  | G | 356(70.63) | 420(70.47) | 1.008(0.777-1.308) | | 0.503 |
| A_L_M_H_ | AA | 90(79.65) | 80(73.39) | ref. | |  |
|  | AG/GG | 23(20.35) | 29(26.61) | 0.705(0.377-1.317) | | 0.173 |
|  | A | 149(28.88) | 148(29.37) | ref. | |  |
|  | G | 367(71.12) | 356(70.63) | 1.024(0.782-1.342) | | 0.459 |
| A_H_M_L_ | AA | 84(73.68) | 70(64.81) | ref. | |  |
|  | AG/GG | 30(26.32) | 38(35.19) | 0.658(0.370-1.168) | | 0.099 |
|  | A | 165(30) | 145(29.59) | ref. | |  |
|  | G | 385(70) | 345(70.41) | 0.981(0.751-1.280) | | 0.47 |
| A_H_M_H_ | AA | 74(67.89) | 91(79.13) | ref. | |  |
|  | AG/GG | 35(32.11) | 24(20.87) | 1.793(0.981-3.278) | | 0.039 |
|  | A | 146(28.97) | 164(29.93) | ref. | |  |
|  | G | 358(71.03) | 384(70.07) | 1.047(0.803-1.366) | | 0.393 |

Note: ^a^Comparing Cases with Controls. ^b^From chi-square test, adjusted for BMI.

A+M meant combined exposure to AFB1 and microcystin-LR. A_L_M_L_ was defined as those with AFB1 lower than median and with MC-LR lower than median, and so on.
